# Assessment of Community Pediatric Providers’ Approach to Children With *Helicobacter pylori*

**DOI:** 10.1097/PG9.0000000000000033

**Published:** 2020-12-09

**Authors:** Nan Du, Corey Scherer, Anthony Porto

**Affiliations:** From the * Yale-New Haven Hospital, New Haven, CT; † Akron Children’s Hospital, Akron, OH.

**Keywords:** *Helicobacter pylori*, antibiotics, pediatric, urease breath test, stool antigen testing, *H. pylori* culture

## Abstract

In 2016, the European and North American Societies for Pediatric Gastroenterology, Hepatology, and Nutrition updated guidelines for management of *Helicobacter pylori* in children, adjusting the previous diagnostic and therapeutic approaches in response to changing epidemiology and antibiotic resistance patterns. In this study, we assessed knowledge, practice habits of *H. pylori* management, and adherence to new guideline recommendations among one state’s community pediatric providers. The majority of the surveyed community pediatric providers are not following the new pediatric guidelines for *H. pylori* management. An evidence-practice gap for diagnosis and treatment of *H. pylori* disease can lead to poor resource utilization, inadequate antibiotic usage, increased antibiotic resistance, and prolonged symptoms in patients. By understanding pediatric provider practices, pediatric gastroenterologists can assist in addressing the gap through development of educational interventions for community pediatric providers.

## Abstract 2

Supplemental Digital Content is available in the text.

**What Is Known**

- Guidelines for the management and diagnosis of *Helicobacter pylori* in children has changed due to rising antibiotic resistance as well as changing epidemiology of the infection.
- Although the *H. pylori* guidelines were updated in 2016, there is limited data on their adoption and implementation among community pediatricians.

**What Is New**

- In this study, we found that a majority of the providers surveyed had not adopted the updated diagnostic and treatment guidelines for *H. pylori*.
- Over a third of the respondents reported incorrectly testing patients for *H. pylori* while they were taking proton pump inhibitors.
- Only one in 5 correctly identified the appropriate antibiotic dosing for treatment of *H. pylori* infection in children.

*Helicobacter pylori* is a gram-negative spiral bacterium that typically causes infection in early childhood around the ages of 5 to 10 (1). The prevalence of *H. pylori* in children varies in the world from 2.5% to 34.6% and has been fluctuating rapidly (2). The clinical presentation, need for diagnostic testing, and treatment in children is significantly different from adults where often they present with gastroduodenal ulcer or gastric cancer. Also, unlike in adult guidelines, testing for *H. pylori* is not recommended for children presenting with functional abdominal pain (3,4). The majority of children infected with *H. pylori* are asymptomatic (5). Recent literature has suggested that *H. pylori* infection may convey immunologic benefits later in life (6). The accuracy of noninvasive diagnostic testing in pediatrics is also highly variable.

In 2016, the European and North American Societies for Pediatric Gastroenterology, Hepatology, and Nutrition (ESPGHAN/NASPGHAN) updated guidelines for management of *H. pylori* in children due to the changing epidemiology of *H. pylori* infection and decreasing efficacy of treatment (1). There has been increasing resistance to antibiotics and decreasing efficacy of *H. pylori* eradication in young children. The updated guidelines emphasize the importance of diagnostic testing with endoscopy and performance of *H. pylori* culture to help guide medical therapy. The former “test and treat” strategy has been discouraged. When bacterial culture and sensitivity are not available, empiric therapy with high-dose amoxicillin, metronidazole, and proton pump inhibitor (PPI) is recommended. In addition, the guidelines include a noninvasive test of cure, either a stool antigen or urea breath test, to be performed at least 4 weeks after completion of treatment.

We assessed the knowledge and practice habits of *H. pylori* management among the community pediatric providers in one state and how they adhered to new guidelines recommendations. We hypothesized that at least 20% of the community pediatricians would not be consistently following guideline recommendations.

## METHODS

This study followed a one-time cross-sectional observational study design. Subjects included Connecticut primary care pediatric practitioners affiliated with one tertiary care center and/or affiliated with the Connecticut American Academy of Pediatrics chapter. We developed an electronic survey using an online form through Qualtrics and distributed it through an email list-serve. A questionnaire previously utilized to assess knowledge of celiac guidelines in community pediatricians guided survey development (7). Content was based on recent major changes to the *H. pylori* guidelines. The survey questions addressed each component of the new guidelines: (1) diagnostic testing, (2) first-line treatment choice, (3) antibiotic dosing, and (4) preferred test of cure (Appendix A, Supplemental Digital Content 1, http://links.lww.com/PG9/A7). Prior to finalizing the survey, we solicited iterative feedback from a panel comprised of general pediatricians including our Associate Chief of Clinical Services and several pediatric gastroenterologists. An email survey link was distributed to the providers from July 2019 to September 2019, with 2 remainder emails sent about 1 month later in October 2019. Providers voluntarily completed the anonymous online form. Institutional review board approval through exemption was obtained (Human Investigation Committee 200022326). We analyzed the results with descriptive statistics in excel.

## RESULTS

### Demographics

A total of 101 providers completed the questionnaire. Seventy percent of the providers were female. The majority (91%) of responders identified themselves as physicians. Most (83%) had greater than 10 years of experience. Fifty-one percent of the providers reported that they diagnosed *H. pylori* within the practice (Table 1).

**Table 1.** Pediatric provider respondent characteristics

Demographics	n (%)
Providers	101 (100)
Physicians	92/101 (91)
Female	71/101 (70.3)
Years in Practice	
<5 yr	7/101 (6.9)
5–10 yr	10/101 (9.9)
>10 yr	84/101 (83.2)
Diagnose *Helicobacter pylori* in practice	42/83 (51)

### Diagnostic Testing

Of the subset who reported experience diagnosing *H. pylori*, 56% of the providers used stool antigen testing, 44% used urea breath testing, and 17% used blood serology as a diagnostic test. Providers could choose more than one test. Most providers (62%) reported that they were somewhat confident or very confident in their choice of diagnostic test. About a third (34%) of providers reported performing a *H. pylori* stool antigen diagnostic test while their patients were on PPIs. If noninvasive testing was positive, 26% of the providers reported that they referred greater than 95% of their patients to a pediatric gastroenterologist for management. Forty-four percent of the providers referred atleast 50% of their patients to a pediatric gastroenterologist for management. The rest were presumably treated by their primary provider, although were not directly asked on the survey (Table 2).

**Table 2.** *Helicobacter pylori* management and diagnosis

Providers’ survey responses	n (%)
Preferred testing for Helicobacter pylori*	100
Blood serology	17 (17)
Stool antigen testing for *H. pylori*	57 (56.4)
Urea breath test	44 (43.6)
Urine serology	0 (0)
If performing stool antigen test, how often would patients be on PPI?	53
<5%	24 (45.2)
5–25%	9 (16.9)
25–50%	3 (5.7)
50–75%	3 (5.7)
75–95%	0 (0)
>95%	3 (5.7)
Not relevant	11 (20.7)
Frequency patient is referred to pediatric gastroenterologist for *H. pylori*	93
<5%	23 (24.7)
5–25%	16 (17.2)
25–50%	9 (9.7)
50–75%	10 (10.8)
75–95%	7 (7.5)
>95%	24 (25.8)
Not relevant	4 (4.3)
Treatment for *H. pylori*	93
Amoxicillin and clarithromycin	43 (46)
Amoxicillin and metronidazole	26 (28)
Clarithromycin and metronidazole	13 (14)
Metronidazole and tetracycline	1 (14.9)
Clarithromycin	4 (4.3)
Amoxicillin	3 (3.2)
Metronidazole	2 (2.2)
Provider’s assessment of cure*	94
Repeat blood serology	3 (3.0)
Based on resolution of symptoms	64 (63.4)
Repeated stool antigen testing	25 (24.8)
Repeat urine serology	0 (0)
Repeat urease breath testing	23 (22.8)

*Able to select multiple answers.

PPI = proton pump inhibitor.

### Treatment and Assessment of Cure

Twenty-eight percent of pediatric providers correctly stated that first-line therapy contained metronidazole and amoxicillin. Out of the providers who selected the correct first-line antibiotic therapy, 20% selected the correct amoxicillin dose. The majority selected incorrect first-line antibiotic dosing responses when antibiotic sensitivities were not known. Sixty percent of providers selected first-line therapy that at least contained clarithromycin: 14% of providers selected clarithromycin and metronidazole, while 46% of the providers selected amoxicillin and clarithromycin as first-line therapy treatment. Thirty-four percent of the providers who chose the correct regimen stated that they were at least somewhat confident on their answers and 67% of the providers who choose the incorrect regimen reported that they were at least somewhat confident on their answers.

In assessing test of cure, 42% selected either urease breath test or stool antigen testing. Approximately 63% of pediatric providers assessed cure by resolution of symptoms (Table 2). The most commonly used resources reported by pediatric providers were UpToDate (45%) and Redbook (31%).

## DISCUSSION

*H. pylori* diagnosis, management, and eradication in children continue to remain a challenge as evident by variation in provider answers. The 2016 ESPGHAN/NASPGHAN recommendations were proposed due to decreased efficacy of *H. pylori* eradication and increased antibiotic-resistant strains (1). Recognition of the evidence-practice gap among community pediatric providers for the approach of diagnosis and treatment of *H. pylori* is vital to improving clinical practice (Appendix B, Supplemental Digital Content 2, http://links.lww.com/PG9/A8).

### Diagnosis

In our study, a majority of the providers were incorrectly utilizing the now disproven “test and treat” strategy for *H. pylori* infection in children and doing so through nonguideline concordant noninvasive testing. Noninvasive testing is inappropriate given the low prevalence of primary infection among children born and raised in North America (8,9). Diagnostic testing for *H. pylori* with noninvasive test for children experiencing pain or nausea and treating in case of positive result is not recommended as it can be found incidentally (6,10). *H. pylori* infection typically does not play a role in functional abdominal pain disorders and treatment will unlikely provide symptom relief or benefit (10). Furthermore, infection with *H. pylori* in an otherwise asymptomatic patient has also been associated with potentially immunologic benefits later in life including decrease risk of Barrett’s esophagus, celiac disease, and inflammatory bowel disease (11–13). *H. pylori* treatment should only be started if a patient is symptomatic with presence of ulceration on endoscopy. In those using noninvasive diagnostic testing, a third of the providers did not have their patients off of a PPI at time of testing for *H. pylori*. Calvet (14) showed that PPI therapy can suppress *H. pylori* replication and hence can give false negative diagnostic test results. Patients should be off PPI for at least 2 weeks to reduce the chance of false negative testing (15). It is important for pediatric providers to understand that *H. pylori* serology is no longer a viable diagnostic test and can lead to unnecessary treatment.

### Treatment

Beyond the deviation from guidelines with the persistence of noninvasive testing among community pediatric practices, we found only one in 5 pediatric providers were treating their patients with the evidence-based guideline recommended type and dosing of antibiotics. Similar to a study of pediatricians in Israel, we found that most participants prescribed clarithromycin, even though there is rising clarithromycin resistance to *H. pylori* in community (16). Clarithromycin resistance to *H. pylori* in the United States is estimated between 23% and 46% (17). This is a major inconsistency with the new guidelines and could continue to perpetuate the local antibiotic resistance to *H. pylori* strains. Dosing of antibiotics is also important as almost a fifth of the providers who chose the correct antibiotic of amoxicillin selected incorrect dosing. Electronic order sets with weight-based dosing already preassigned may assist in decreasing future errors (18). Providers should be aware that when ordering combination medications such as Prevpac (Lansoprazole, Amoxicillin, and Clarithromycin) for older children that their patients may get inadequate dosing of the medication.

Only about half of the providers reported referring patients to pediatric gastroenterology for testing and treatment, which may increase risk of treatment failure as the majority of providers only assessed cure of *H. pylori* by resolution of symptoms. These patients should be referred and receive an endoscopy before starting treatment. In settings where endoscopy and pediatric gastroenterology are not readily available, such as during the coronavirus disease pandemic, it is imperative that pediatricians be aware of appropriate dosing for antibiotics.

### Test of Cure

A majority of our surveyed pediatric providers incorrectly assessed cure by resolution of symptoms. Even when children are asymptomatic after treatment, it is imperative that test of cure be performed. For those that performed test of cure, the majority of providers appropriately chose the evidence-based guideline indicated tests, either stool antigen or urease breath test. Test of cure should be performed to not only confirm *H. pylori* eradication in patients but can also potentially inform on community antibiotic resistance patterns (19). Test of cures should be performed at least 2 weeks after stopping the PPI and at least 4 weeks after completion of the antibiotic treatment (1).

Overall, we have shown that community pediatricians have poor adherence to the recent *H. pylori* 2016 guidelines. The most commonly used resource for our pediatricians was UpToDate. We hypothesize that one future intervention would be to create a pediatric-focused *H. pylori* treatment page on UpToDate. Currently, while searching for “*H. pylori* in children/pediatrics” on UpToDate, the search results were limited to solely link to guidelines. While the recent *JPGN* guidelines were linked, many of the other links were composed of adult management guidelines, which are vastly different from pediatric guidelines. There are many other potential reasons for the evidence-practice gap, and understanding barriers to unlearning and implementing new knowledge is a novel research area alone (20).

There are several limitations to our study. Our study is an observational study from a single state. Although it includes greater than 100 pediatric providers in one region, it may not reflect the national clinical practice and knowledge of all providers practicing in North America. We did not compare with other types of providers, including pediatric gastroenterologists. In addition, we do not have knowledge of the exact percentage of respondents. The survey did not have any mechanisms to prevent respondents from consulting outside sources, and responses may not be reflective of clinical practice. Furthermore, the survey also relied on self-respondent questionnaires that may have recall and response biases.

Future work will focus on improvement of systems, including dissemination of new guidelines, educational outreach, and integration of practice protocols to address barriers to learning and implementing new knowledge and to ensure official adherence to *H. pylori* guidelines. As pediatric gastroenterologists, we can contribute to closing this gap by providing educational outreach and work with our primary care colleagues to identify impactful informative interventions. We provided survey respondents an informative PowerPoint (Appendix C, Supplemental Digital Content 3, http://links.lww.com/PG9/A9) after completion of survey and provided community outreach to the providers.

## CONCLUSIONS

Although new guidelines for *H. pylori* diagnosis and management have been in place since 2016, the majority of surveyed community providers have not yet adopted them in full. An evidence-practice gap for use of noninvasive testing for diagnosis of *H. pylori* disease can lead to poor resource utilization, inadequate antibiotic prescriptions, worsening antibiotic resistance, and prolonged symptoms in patients. Opportunities exist for improved education interventions and communication with providers on appropriate antibiotic dosing as well as appropriate follow-up of patients with *H. pylori*. As pediatric gastroenterologists, we have a role to play in ensuring our primary care colleagues are informed on new guidelines modifications.

## Acknowledgments

The authors thank Dr Gregory Germain for his assistance in distributing the survey. The authors also thank Dr Julia Rosenberg for her critical review of the article.

## Supplementary Material


