# Supplementary material for: Assessment of Community Pediatric Providers’ Approach to Children With Helicobacter pylori
Source: JPGN Rep. 2020 Dec 9;2(1):e033. doi: 10.1097/PG9.0000000000000033 (PMC10191512; doi:10.1097/PG9.0000000000000033)
Supplement: Supplementary file 1 [file pg9-2-e033-s001.pdf]

***H.pylori* Management/ Diagnosis Study**

In 2016, the European and North American Societies of Pediatric Gastroenterology and Nutrition published new guidelines on the management of *H.pylori* infection in children. The aim of this study is to investigate the general practitioner's screening practices and management of a child with *H.pylori* disease. After you complete the survey, we will provide you with a summary of the *H.pylori* management recommendation. Participation in the survey is anonymous and completely voluntary. It should take you less than 5 minutes to complete the survey. Thank you for your participation.

1) What kind of provider are you?
   - ☐ Physician
   - ☐ Advance Practice Provider
2) How many years have you been in practice?
   - ☐ <5 years
   - ☐ 5-10 years
   - ☐ >10 years
3) Which gender do you identify with?
   - ☐ Female
   - ☐ Male
   - ☐ Other
4) When you are considering a diagnosis of *H.pylori,* what testing do you perform?
   - ☐ Blood serology
   - ☐ Urine serology
   - ☐ Stool antigen testing for *H.pylori*
   - ☐ Urea Breath test
   - ☐ None of the above
5) How confident are you in diagnostic tests for *H.pylori*?
   - ☐ Not Confident at All
   - ☐ Minimally Confident
   - ☐ Neutral
   - ☐ Somewhat Confident
   - ☐ Extremely Confident
6) (ONLY GIVEN IF STOOL ANTIGEN CHECKED OFF) If you perform a H. pylori stool antigen test, how often is your patient taking a proton pump inhibitor?
   - ☐ <5%
   - ☐ 5-25%
   - ☐ 25-50%
   - ☐ 50-75%
   - ☐ 75-95%%
   - ☐ >95%
7) If you diagnosis *H.pylori,* how often do you refer to GI?
   - ☐ <5%
   - ☐ 5-25%
   - ☐ 25-50%
   - ☐ 50-75%
   - ☐ 75-95%%
   - ☐ >95%
8) If you diagnose and treat *H.pylori*, which of the following antibiotics do you typically use for first line treatment?(Choose 2)
   - ☐ Amoxicillin
   - ☐ Metronidazole
   - ☐ Clarithromycin
   - ☐ Levofloxacin
   - ☐ Tetracycline
   - ☐ Ceftriaxone
   - ☐ Cefdinir
9) How confident are you in this answer?
   - ☐ Not Confident at All
   - ☐ Minimally Confident
   - ☐ Neutral
   - ☐ Somewhat Confident
   - ☐ Extremely Confident
10) What resources or references do you use to guide selection of medications and dosing?
    - ☐ UpToDate
    - ☐ Google
    - ☐ Redbook
    - ☐ Medical Society Guidelines
    - ☐ Other________________
11) When H. pylori sensitivities are not known, what is the recommended dose of amoxicillin in the treatment of *H.pylori* in a 20 kg child?
    - ☐ 250 mg twice a day
    - ☐ 500 mg twice a day
    - ☐ 750 mg twice a day
    - ☐ 1500 mg twice a day
12) How confident are you in this answer?
    - ☐ Not Confident at All
    - ☐ Minimally Confident
    - ☐ Neutral
    - ☐ Somewhat Confident
    - ☐ Extremely Confident
13) How do you assess cure of *H.pylori*?
    - ☐ Based on resolution of symptoms
    - ☐ Repeat stool antigen testing
    - ☐ Repeat blood serology
    - ☐ Repeat urine serology
    - ☐ Repeat urease breath testing
