# Supplementary material for: Assessment of Community Pediatric Providers’ Approach to Children With Helicobacter pylori
Source: JPGN Rep. 2020 Dec 9;2(1):e033. doi: 10.1097/PG9.0000000000000033 (PMC10191512; doi:10.1097/PG9.0000000000000033)
Supplement: Supplementary file 2 [file pg9-2-e033-s002.pdf]

Appendix B: Summary of Providers' Nonadherence to 2016 NASPGHAN *H. pylori* Guidelines

- Majority of providers perform noninvasive "test and treat" strategy
  - *H. pylori* treatment should only be started if patient is symptomatic with presence of ulceration on endoscopy.
- Patients should be diagnosed with endoscopy before starting treatment:
  - Non-invasive testing should not be used to diagnose *H. pylori*
- Providers often do not stop proton pump inhibitor (PPI) therapy at time of testing for *H. pylori*
  - Children should be off of a PPI for at least 2 weeks prior to testing
- Many providers prescribe clarithromycin as part of triple medical therapy
  - Due to increased clarithromycin resistance in the United States of America, clarithromycin should only be part of medical therapy in children with *H. pylori* where sensitivity to clarithromycin is known
- One of five providers who chose the correct antibiotic had selected the incorrect dosing of the antibiotic
  - High dose amoxicillin, up to 3 g per day, is indicated in the setting of unknown sensitivity as part of triple therapy with metronidazole and PPI.
- More than half of providers reported diagnosing *H. pylori* in practice
  - Pediatric gastroenterologists should be involved in diagnosis and treatment of *H. pylori*
- Majority of providers (>60%) incorrectly assessed cure by resolution of symptoms
  - Test of cure should be performed 2 weeks after stopping the PPI and at least 4 weeks after completion of the antibiotic treatment
