# Supplementary material for: Assessment of Community Pediatric Providers’ Approach to Children With Helicobacter pylori
Source: JPGN Rep. 2020 Dec 9;2(1):e033. doi: 10.1097/PG9.0000000000000033 (PMC10191512; doi:10.1097/PG9.0000000000000033)
Supplement: Supplementary file 3 [file pg9-2-e033-s003.pdf]

Review of Current Management and Treatment Guidelines for *H. Pylori* in Children

Nan Du
Anthony Porto

H. pylori

- Helicobacter pylori(*H. pylori): * gram negative spiral bacterium with infection
- Majority of children infected with *H. pylori* are asymptomatic
- Infection in early childhood may have possible benefits later in life
- Management and Eradication of *H. pylori* has become increasingly challenging due to clarithromycin resistance, poor adherence to medical therapy, and low gastric pH

2016 NASPGHAN Updated Guidelines for H. Pylori

- Updated due to the changing epidemiology of H. pylori infection and decreasing efficacy of treatment
- Emphasize the importance of invasive diagnostic testing with endoscopy and performance of H. pylori culture to help guide medical therapy
- Former test and treat strategy was discouraged and the regimen for empiric treatment was changed to high-dose amoxicillin, metronidazole and proton pump inhibitor (PPI).

If you were considering a diagnosis of *H. pylori*, what testing would you perform?

A) Blood serology
B) Urine serology
C) Stool antigen testing for *H. pylori*
D) Urea breath test
E) None of the above

Diagnostic Testing: Guidelines recommend endoscopy biopsy based testing to evaluate for underlying cause of symptoms. Stool antigen and breath test are not recommended to diagnose *H. pylori* since presence of *H. pylori* may be incidental and not related to symptoms. Culture of biopsy can also help guide therapy

If you diagnosis and treat *H. pylori*, which of the following antibiotics do you typically use for first line treatment?(Chose 2)

- Amoxicillin
- Metronidazole
- Clarithromycin
- Levofloxacin
- Tetracycline
- Ceftriaxone
- Cefdinir

First line treatment should include a PPI as well as the following two antibiotics: ***Amoxicillin and Metronidazole***. Clarithromycin should only be used if the strain is known to be susceptible

When *H. pylori* sensitivities are not known, what is recommended dose of amoxicillin in the treatment of *H. pylori* in a 20 kg child?

- 250 mg twice a day
- 500 mg twice a day
- 750 mg twice a day
- 1500 mg twice a day

Patient is 20 kg which is between 15-24kg, which would have amoxicillin 750mg BID. High dose amoxicillin is needed since sensitivities are unknown

Susceptibility-based treatment for *H. pylori*

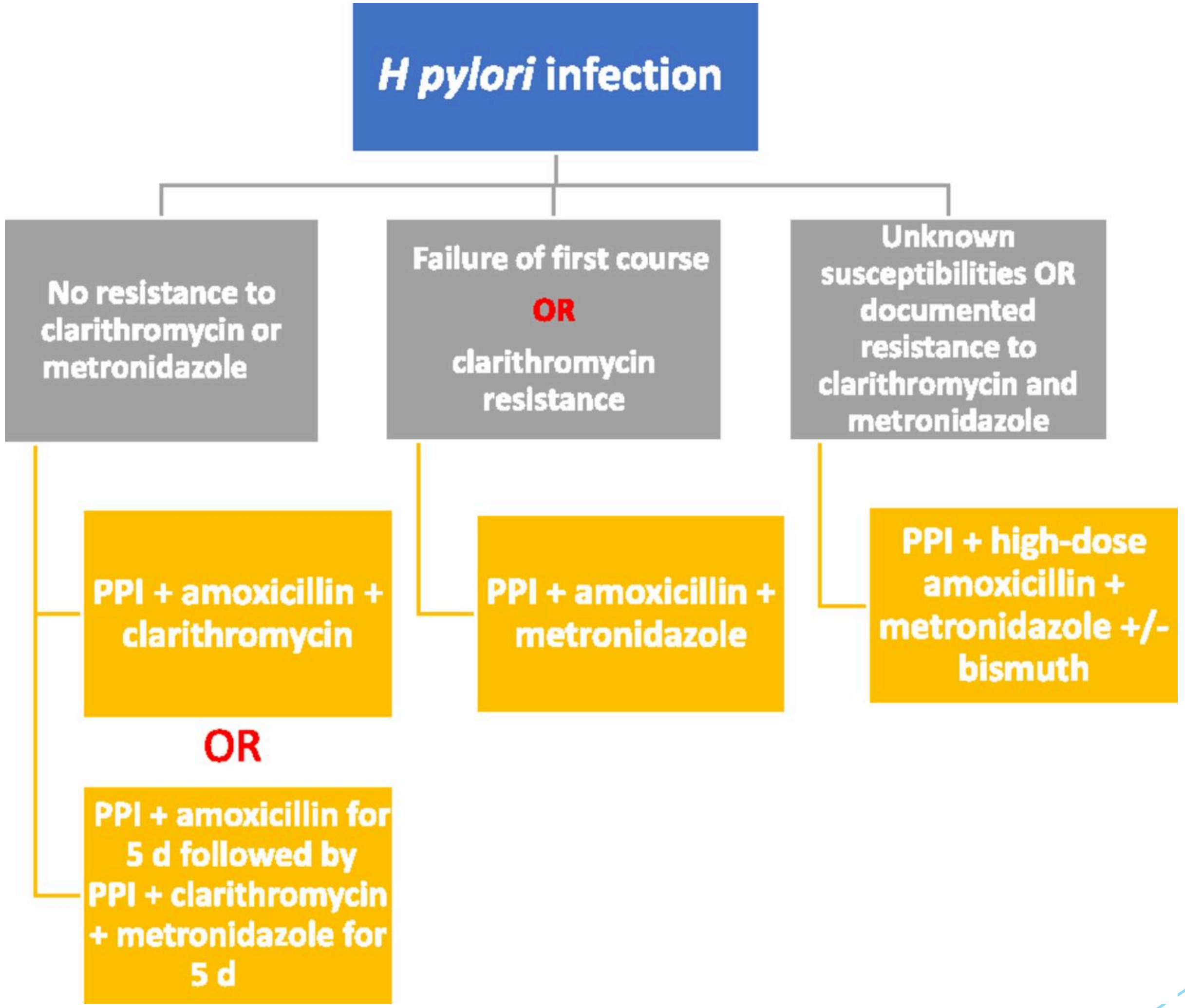

Desiree Sierra et al. Pediatrics in Review 2018;39:542-549

©2018 by American Academy of Pediatrics

PediatricsinReview
AN OFFICIAL JOURNAL OF THE AMERICAN ACADEMY OF PEDIATRICS

Weight-based doses for *H. pylori* treatment if sensitivities are known

PPI	• 15 – 24 kg give 20 mg BID<br>• 25 – 34 kg give 30 mg BID<br>• ≥35 kg give 40 mg BID
Amoxicillin	• 15 – 24 kg give 500 mg BID<br>• 25 – 34 kg give 750 mg BID<br>• ≥35 kg give 1,000 mg BID
Clarithromycin	• 15 – 24 kg give 250 mg BID<br>• 25 – 34 kg give 500 mg in am and 250 mg in pm<br>• ≥ 35 kg give 500 mg BID
Metronidazole	• 15 – 24 kg give 250 mg BID<br>• 25 – 34 kg give 500 mg in am and 250 mg in pm<br>• ≥35 kg give 500 mg BID

Desiree Sierra et al. Pediatrics in Review 2018;39:542-549

PediatricsinReview
AN OFFICIAL JOURNAL OF THE AMERICAN ACADEMY OF PEDIATRICS

©2018 by American Academy of Pediatrics

Weight-based doses for *H. pylori* treatment if sensitivities are not known

TABLE 4. High dosing regimen for amoxicillin

Bodyweight range, kg	Morning dose, mg	Evening dose, mg
15–24	750	750
25–34	1000	1000
>35	1500	1500

How would you assess cure of *H. pylori*?

- Based on resolution of symptoms
- Repeat stool antigen testing
- Repeat blood serology
- Repeat urine serology
- Repeat urease breath testing

Test of Cure: Urease breath test OR Stool antigen testing should be performed at least 4 weeks after completion of treatment

Summary of New Guideline Recommendations

**Diagnosis**		
**Positive H.pylori culture on endoscopy biopsy**		
**First Line Treatment**		
**Antimicrobial Sensitivity**	Suggested Regimen	Amoxicillin Dosing
**Susceptible Unknown**	PPI-AMO-MET 14 days	High-dose or bismuth-based
**Test of Cure**		
**To be completed 4 weeks after completion of therapy**	• ^{13}C-urea breath test<br>or<br>• 2-step monoclonal stool antigen test	

PPI Use in *H. Pylori* Treatment

- Younger children need a higher PPI dose per kg bodyweight compared to adolescents and adults to obtain sufficient acid suppression
- PPI should be given 15 minutes before meal
- Esomeprazole and Rabeprazole are less susceptible to degradation by rapid metabolizers with CYP2C19 genetic polymorphism and may be preferred when available
